# Myocardial inflammation, injury and infarction during on-pump coronary artery bypass graft surgery

**DOI:** 10.1186/s13019-017-0681-6

**Published:** 2017-12-16

**Authors:** Shirjel R. Alam, Colin Stirrat, Nick Spath, Vipin Zamvar, Renzo Pessotto, Marc R. Dweck, Colin Moore, Scott Semple, Ahmed El-Medany, Divya Manoharan, Nicholas L. Mills, Anoop Shah, Saeed Mirsadraee, David E. Newby, Peter A. Henriksen

**Affiliations:** 1 BHF Centre for Cardiovascular Science, University of Edinburgh, The Chancellor’s Building, Little France Crescent, Edinburgh, EH16 5SA UK; 2 NHS Lothian, Edinburgh, UK; 3 Department of Cardiothoracic Surgery, Edinburgh Heart Centre, Edinburgh, UK; 4 Clinical Research Imaging Centre, University of Edinburgh, Edinburgh, UK

**Keywords:** CABG, Troponin, Inflammation, Type 5, Myocardial infarction

## Abstract

**Background:**

Myocardial inflammation and injury occur during coronary artery bypass graft (CABG) surgery. We aimed to characterise these processes during routine CABG surgery to inform the diagnosis of type 5 myocardial infarction.

**Methods:**

We assessed 87 patients with stable coronary artery disease who underwent elective CABG surgery. Myocardial inflammation, injury and infarction were assessed using plasma inflammatory biomarkers, high-sensitivity cardiac troponin I (hs-cTnI) and cardiac magnetic resonance imaging (CMR) using both late gadolinium enhancement (LGE) and ultrasmall superparamagnetic particles of iron oxide (USPIO).

**Results:**

Systemic humoral inflammatory biomarkers (myeloperoxidase, interleukin-6, interleukin-8 and c-reactive protein) increased in the post-operative period with C-reactive protein concentrations plateauing by 48 h (median area under the curve (AUC) 7530 [interquartile range (IQR) 6088 to 9027] mg/L/48 h). USPIO-defined cellular myocardial inflammation ranged from normal to those associated with type 1 myocardial infarction (median 80.2 [IQR 67.4 to 104.8] /s). Plasma hs-cTnI concentrations rose by ≥50-fold from baseline and exceeded 10-fold the upper limit of normal in all patients. Two distinct patterns of peak cTnI release were observed at 6 and 24 h. After CABG surgery, new LGE was seen in 20% (*n* = 18) of patients although clinical peri-operative type 5 myocardial infarction was diagnosed in only 9% (*n* = 8). LGE was associated with the delayed 24-h peak in hs-cTnI and its magnitude correlated with AUC plasma hs-cTnI concentrations (*r* = 0.33, *p* < 0.01) but not systemic inflammation, myocardial inflammation or bypass time.

**Conclusion:**

Patients undergoing CABG surgery invariably have plasma hs-cTnI concentrations >10-fold the 99th centile upper limit of normal that is not attributable to inflammatory or ischemic injury alone. Peri-operative type 5 myocardial infarction is often unrecognised and is associated with a delayed 24-h peak in plasma hs-cTnI concentrations.

**Electronic supplementary material:**

The online version of this article (doi: 10.1186/s13019-017-0681-6) contains supplementary material, which is available to authorized users.

## Background

Approximately 400,000 coronary artery bypass graft (CABG) operations are performed each year in the United States of America [1]. Cardiopulmonary bypass (CPB) during these procedures is known to induce systemic and myocardial inflammation [2, 3]. Inflammatory cytokines such a tumour necrosis factor alpha (TNF-α) depress cardiac function after CPB [4], whereas elevations of interleukin(IL)-6 and IL-8 are proportional to levels of myocardial injury and apoptosis [5]. Elevated cardiac troponin concentrations have been identified in up to 100% of patients undergoing CABG [6]. However, corresponding cardiac magnetic resonance (CMR) evidence of myocardial necrosis is evident in only 28% of patients, suggesting that troponin release may reflect reversible injury resulting from other processes such as inflammation or ischaemia-reperfusion injury rather than infarction [7]. The identification of clinically relevant peri-operative myocardial injury and infarction can be challenging but is important because it predicts short, medium and long-term mortality [8, 9].

The universal definition of myocardial infarction defines procedural (type 5) myocardial infarction as a > 10-fold elevation above the 99th centile of cardiac troponin within 48 h of CABG surgery accompanied by new pathological Q waves or left bundle branch block, angiographically documented new graft or new native coronary artery occlusion, or imaging evidence of new loss of viable myocardium or new regional wall motion abnormality [10]. Elevation of plasma cardiac troponin concentration is required but in the absence of these other clinical features, is insufficient to make the diagnosis of type 5 myocardial infarction.

The latest generation of high-sensitivity cardiac troponin I (cTnI) assays have been widely adopted since the introduction of the third universal definition of myocardial infarction. Our group has contributed to work demonstrating the ability of these assays to define normal plasma concentrations in healthy populations and to identify at-risk patients presenting with chest pain and very small elevations in cTnI concentration that would pass undetected by older contemporary assays [11]. This increased sensitivity may have consequences for the identification and diagnosis of myocardial infarction in patients undergoing CABG surgery.

Using a comprehensive panel of blood and imaging biomarkers, we here explored the relationship between perioperative myocardial inflammation, injury and infarction in order to characterise and to diagnose type 5 myocardial infarction with high-sensitivity cTnI assays.

## Methods

The Elafin Myocardial Protection from Ischemia Reperfusion (EMPIRE, ISRCTN 82061264) randomized controlled clinical trial investigated the effect of Elafin, a neutrophil elastase inhibitor, on myocardial injury during on-pump CABG surgery [12]. This single centre clinical trial was performed with the approval of the national research ethics committee (11/MRE00/5), in accordance with the Declaration of Helsinki (2000), under a Clinical Trial Authorization (27,586/0015/001–0001) from the Medicine and Healthcare products Regulatory Authority (MHRA, United Kingdom), and the written informed consent of all participants. The authors had no conflicts on interests.

### Coronary artery bypass graft surgery

The EMPIRE study recruitment, cardiac anesthesia, surgery and protocols have been described previously [12]. Briefly, patients referred for elective CABG surgery were recruited at Edinburgh Heart Centre between June 2011 and September 2013. Key exclusion criteria included recent myocardial infarction (within 1 month of surgery), emergency or concomitant valve surgery, significant renal impairment (estimated glomerular filtration rate < 40 mL/min), severe respiratory disease, severe left ventricular impairment (ejection fraction <40%), contraindication to magnetic resonance scanning and on-going treatment for chronic inflammatory disease. Surgical approach was via a median sternotomy and cardiopulmonary bypass was started after heparin administration with a non-pulsatile flow and a membrane oxygenator. Cardioprotection was provided by cold-blood cardioplegia (1:4), which was administered anterogradely, after cross-clamping the aorta, into the coronary arteries or by cross clamp fibrillation. Where possible the left internal mammary artery was used for grafting. Other conduits were chosen from saphenous vein, radial artery or the right internal mammary artery.

### Electrocardiogram

An ECG was performed prior to surgery, and then immediately post-surgery, 24 h, 48 h and at discharge. Additional ECGs were performed only if clinically indicated. Ischemia or infarction by ECG was defined as at least 2 mm ST deviation in the chest leads or 1 mm in the limb leads, development of new pathological Q waves or new bundle branch block. Other ECG categories included T-wave inversion (TWI) with no signs of ischemia or infarction, concave ST elevation with or without PR segment depression and no changes.

### Blood biomarkers

Blood samples were taken at baseline (time 0 h, skin incision) and at 2, 6, 24 and 48 h post-operatively. Stored plasma samples were analysed using the ARCHITECTSTAT high-sensitive troponin I assay (Abbott Laboratories, Abbott Park, IL) with a limit of detection of 1.2 ng/L and the inter-assay CV <10% at 4.7 ng/L. The 99th centile upper reference limit (URL) were 34 ng/L (men) and 16 ng/L (women) [13]. Plasma concentrations of high-sensitive C-reactive protein (hsCRP), interleukin (IL)-6, IL-8, tumour necrosis factor (TNF)-α, myeloperoxidase and elastase were quantified using enzyme-linked immunosorbant assays (ELISAs; R&D Systems, U.K.; Elastase ELISA, Cambridge Biosciences, U.K.).

### Cardiac magnetic resonance imaging

Cardiac magnetic resonance (CMR) imaging scans were performed at baseline (up to 6 weeks before surgery) and as soon as clinically feasible from the fifth post-operative day. After the post-operative CMR scan, patients received an intravenous infusion of ultrasmall superparamagnetic particles of iron oxide (USPIO; ferumoxytol, Advanced Magnetics Inc., Cambridge, MA) at a dose of 4 mg/kg. A third CMR scan was performed 24 h after USPIO infusion. All patients completed scanning within 14 days of surgery. Quantification of left ventricular mass, ejection fraction and late gadolinium enhancement infarct size were determined using established protocols and dedicated cardiac analysis software by two trained independent blinded observers. Late enhancement analysis was performed using QMass software (Medis medical imaging systems, Netherlands), allowing quantification of infarct size using the full-width, half-maximum criterion [12].

Patients were scanned using a 3 T Siemens Verio scanner (Siemens Medical, Germany) as described previously [12]. USPIO imaging was performed using established T2*-weighted multi-gradient-echo sequences, with USPIO images quantitatively analysed using a susceptibility gradient mapping post-processing technique previously described [14, 15]. Quantitative analysis of USPIO accumulation was achieved by calculation of T2* relaxation times before and after administration of USPIO [16]. T2*-weighted multi-gradient-echo images for the second and third (post-USPIO) scan were spatially co-registered using ANALYZE software (AnalyzeDirect Software, United States). The four echoes from the range (TE = 2.13, 4.27, 6.41, 8.55 ms) in a multi-echo T2*-weighted sequence were combined to generate a T2* map, using in-house software developed in Matlab (Mathworks, US) as previously described [15]. The inverse of T2*, R2*, was then calculated to assess USPIO uptake and thus a higher R2* value would indicate a higher level of inflammatory cell infiltration. The myocardium was divided into the standard 17-segment model, and a R2* value calculated for each segment [17]. After calculating the increase in R2* value between the second and third scans, a pan-myocardial R2* value calculated from the mean of all segments. Since inflammation in the myocardium was likely to be non-uniform, a mean of the highest three segments was also calculated.

### Data and statistical analysis

All statistical analysis was performed with GraphPad Prism version 4.00 (GraphPad Software, San Diego California USA). We have previously reported [12] that Elafin had no effect on all outcome measures assessed and data from patients receiving placebo and active treatment arms were therefore aggregated. Plasma hs-cTnI, hs-CRP, IL-6, IL-8 and MPO concentrations at each time point were expressed as median [inter-quartile range], mean ± standard error of mean or mean (95% confidence intervals) as appropriate. Forty-eight hour area-under-the-curve (AUC) was calculated for plasma hs-cTnI, hsCRP and cytokines.

The change in mass of infarcted tissue was determined by the difference in LGE from the preoperative and first postoperative CMR scans. This was categorized as increased, no change or reduced according to detection threshold based on inter-observer variability *and* agreement between both independent analysers. The absolute change in ejection fraction from pre to post surgery was calculated and an arbitrary value of 5% change was used to categorize patients into increased, decreased or no change.

Categorical data were compared using the Pearson’s chi-square test. Non-parametric R2* data were analysed with the Mann-Whitney U-test. Multiple comparisons for USPIO uptake were compared using Kruskal-Wallis. If significant, Dunn’s multiple comparison post-test was performed comparing R2* value across groups. Statistical significance was defined as two-sided *P* < 0.05.

## Results

**Fig. 1:**
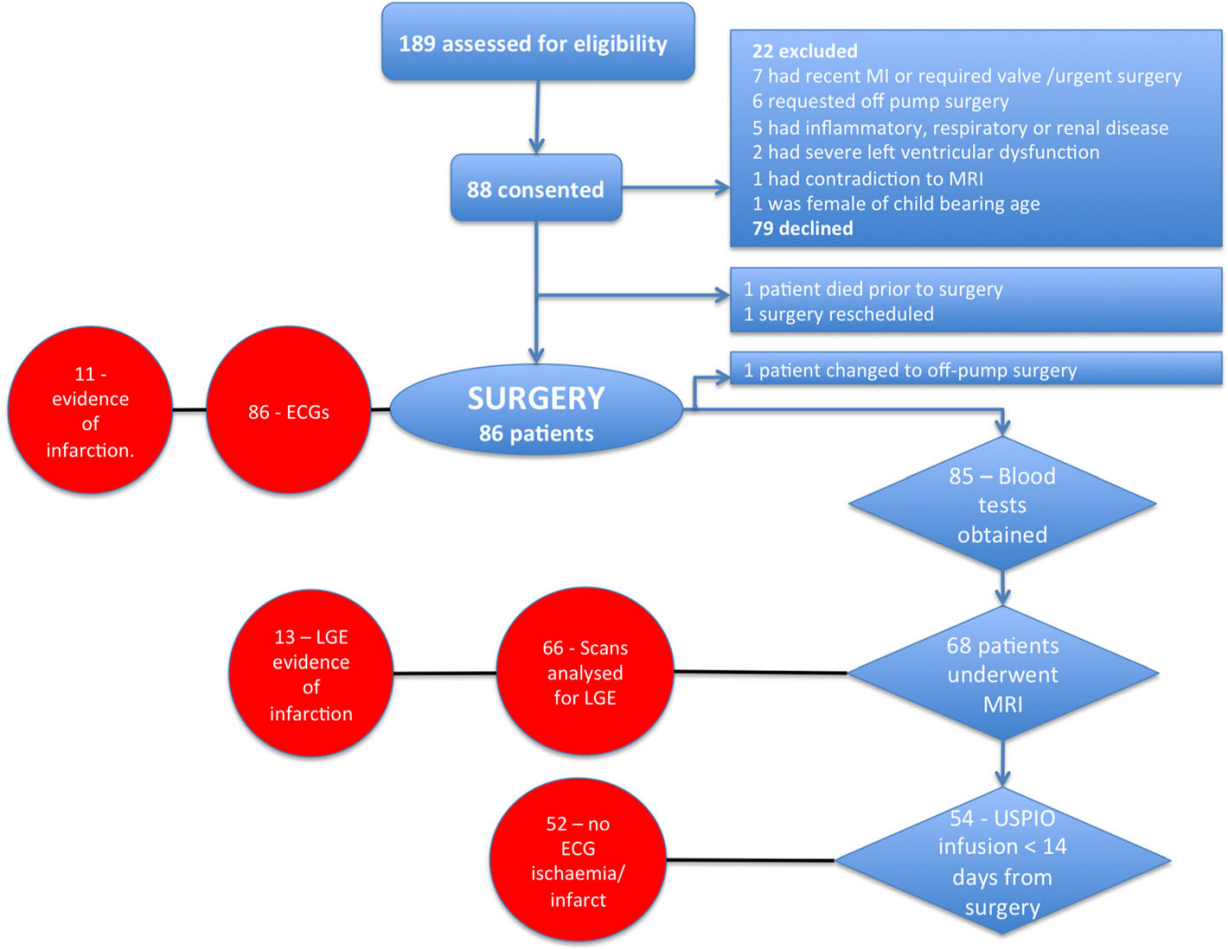
Study flow diagram

Baseline characteristics have been reported according to treatment group previously [12]. Consecutive patients were assessed for eligibility and 53% consented to participate (Fig. 1 and Table 1). Pre-existing (previously unidentified) myocardial infarction was common with 27% (21/79 who underwent pre-surgery MRI) of patients having pre-operative LGE. Two patients died in the early post-operative period from graft failure and cardiac arrest and a further 6 patients were diagnosed with a myocardial infarction by the clinical team based on ECG changes and troponin release giving a total of 8 (9%) patients who were clinically diagnosed with type 5 myocardial infarction. Unrecognised type 5 myocardial infarction was identified in a further 10 patients (total of 18; 21%) with new LGE on CMR.

**Table 1:** Baseline patient characteristics. Mean ± SD or n (%)

Age (years)	63 ± 8
2-vessel coronary disease	23 (26%)
3-vessel coronary disease	64 (74%)
Creatinine (mg/dL)	0.90 ± 0.21
Creatinine Clearance (mL/min)	117 ± 35
Diabetes Mellitus	20 (23%)
Surgeon A	34 (39%)
Surgeon B	53 (61%)
Male Gender	74 (85%)
EuroSCORE	2.4 ± 190
Clinically diagnosed previous MI	32/87 (37%)
Clinical diagnosis of previous MI without LGE	7/79 (9%)
LGE without previous clinically recognized MI	21/79 (27%)
Intra-operative details	
Number of bypass grafts	
One	3
Two	33
Three	36
Four	14
Five	1
Cardiopulmonary bypass time (min)	78 ± 24
Cross clamp time (min)	46 ± 15

Overall 25.3% of patients required intra-aortic balloon pump counterpulsation, inotropes or vasoactive support in the first 48 h, 33% required red cell transfusion, and 31% developed atrial fibrillation prior to discharge (Table 2).

**Table 2:** Clinical outcomes. Mean ± SD or n (%)

	<48 h		In-hospital	
Post-operative complications and outcomes				
Death	0	(0)	2	(0)
Stroke	0	(0)	0	(0)
Clinically diagnosed Myocardial infarction	1	(2.3)	8	(9)
Inotrope or balloon pump support for >24 h	22	(25.3)	22	(25.3)
Red cell transfusion post-op	24	(27.6)	29	(33.3)
Re-operation for bleeding	3	(3.4)	3	(3.4)
Antibiotic administration	6	(6.9)	33	(37.9)
Atrial fibrillation	7	(8)	27	(31)
Peak serum creatinine mg/dL	1.00 ± 0.41		1.03 ± 0.45	
Peak creatinine clearance mL/min	101.9 ± 33.2 101.9 ± 33.2		101.4 ± 30.3 101.4 ± 30.3	

### Humoral and cellular inflammation

#### Humoral inflammation

Systemic inflammatory markers rose following surgery (Table 3, Additional file 1: Figure S1) with IL-6 and MPO peaking at 2 h, and IL-8 and TNF-α continuing to rise at 6 h. Plasma hsCRP concentrations rose steeply at 6 h and continued to rise at 48 h. There was no correlation between cytokines and LGE on CMR. There was weak correlation between AUC hsCRP release and hs-TnI (*r* = −0.27, *p* = 0.01).

**Table 3:** Cardiac troponin and cytokine concentrations

	0 h	2 h	6 h	24 h	48 h	AUC
High-sensitivity cardiac troponin I (ng/l or ng/l/48 h)	3.5 (2, 9.6)	76.5 (35.6, 163.6)	3222 (1413, 5607)	1043 (5344, 2948)	5210 (2302, 1342)	74,480 (35,100, 164,100)
High-sensitivity C-reactive protein (mg/L)	2.00 (0.5, 3.00)	1.00 (0.50, 2.00)	2.00 (1.00, 3.00)	121 (96.0, 148)	236 (182, 279)	7530 (6088, 9027)
Interleukin-6 (pg/mL)	1.80 (1.10, 2.60)	10.0 (5.75, 15.6)	10.5 (8.15, 17.7)			55.8 (43.3, 80.5)
Interleukin-8 (pg/mL)	8.90 (6.28, 11.7)	7.60 (4.95, 16.9)	93.8 (45.9, 148)			240 (121, 411)
Tumour necrosis factor-α (pg/mL)	4.50 (4.50, 7.30)	4.50 (4.50, 11.5)	7.10 (4.50, 14.25)			37.3 (27.0, 66.7)
Myeloperoxidase (ng/mL)	75.6 (45.6, 150)	532 (383, 743)	194 (112, 350)			2175 (1588, 2889)

Median (inter-quartile range)

*AUC* area under the curve

#### Cellular inflammation

**Fig. 2:**
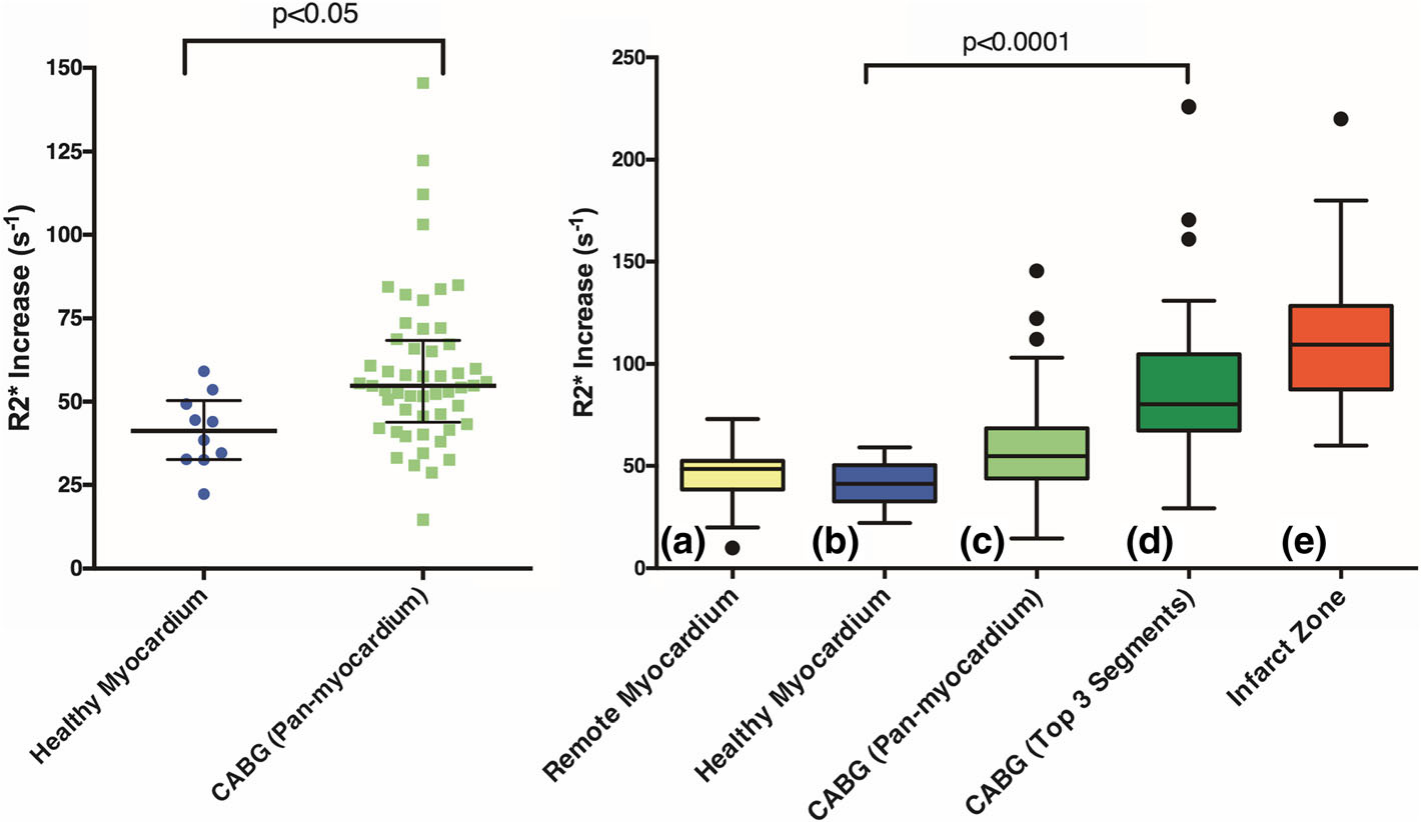
Left – Pan-myocardium R2* increase in healthy volunteers (*n* = 10) and post-coronary artery bypass graft (CABG) surgery. Median and inter-quartile range. Right - Tukey box plot comparing R2* increase in the myocardium of (a) healthy volunteers, (b) patients with acute myocardial infarction (remote from the site of infarction), (c) patients post-CABG surgery (pan-myocardial average), (d) patients post-CABG surgery (average of 3 highest values from 17 segment model), and (e) patients with acute myocardial infarction (site of infarction)

**Fig. 3:**
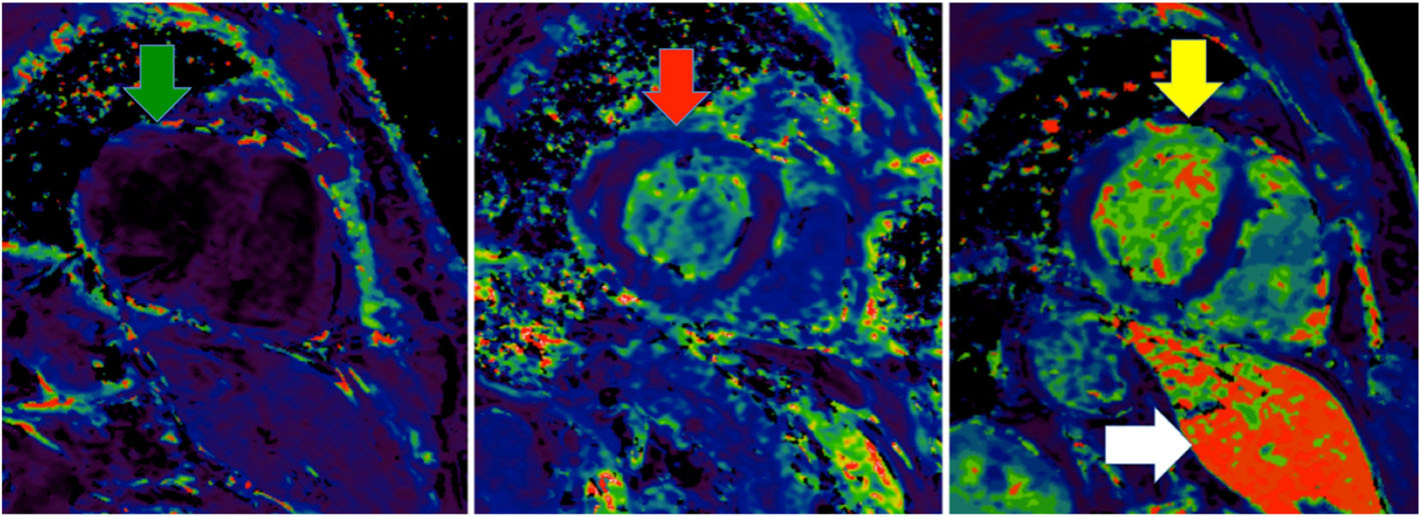
USPIO enhance MRI scans. Myocardium without USPIO enhancement appears indistinct (left image, green arrow). In patients post CABG surgery, USPIO enhanced MRI appears bright globally (centre image, red arrow). In a patient with anterior myocardial infarction, USPIO enhanced MRI has intense intake in the anterior wall (right image, yellow arrow). In the right image the liver is visible which also has intense uptake of USPIO (white arrow)

In total, 54 patients underwent USPIO infusion and CMR scanning within 14 days of surgery. USPIO infusion was well tolerated, with only 1 patient reporting muscle cramps after administration. There were 52 patients with evaluable data for USPIO analysis (Fig. 2) and these were compared with 10 contemporaneous healthy volunteers (mean age 26 ± 5 years, 5 male and 5 female) for reference. Previously published data analysing the infarct zone of patients who had experienced type 1 myocardial infarction are also provided for comparison (Fig. 3) [15]. Patients undergoing CABG had increased pan-myocardial R2* (median 54.8 [interquartile range 43.8 to 68.4] /s versus 41.2 [32.6 to 50.4] /s in healthy volunteers, *p* < 0.05). It was also increased in the highest 3 of the 17 segments (median 80.2 [67.4 to 104.8] /s versus 58.7 [48.8 to 68.6] /s in healthy volunteers, *p* < 0.0001) and was similar to patients who had sustained type 1 myocardial infarction (109.5 [87.5 to 128.3] /s, *p* = 0.41). R2* increase correlated with plasma hsCRP concentrations (Additional file 2: Figure S2; *r* = 0.3, *p* = 0.03) but not with cTnI release (*r* = 0.15, *p* = 0.15) or CPB time (*r* = 0.00, *p* = 0.49).

### Myocardial injury

**Fig. 4:**
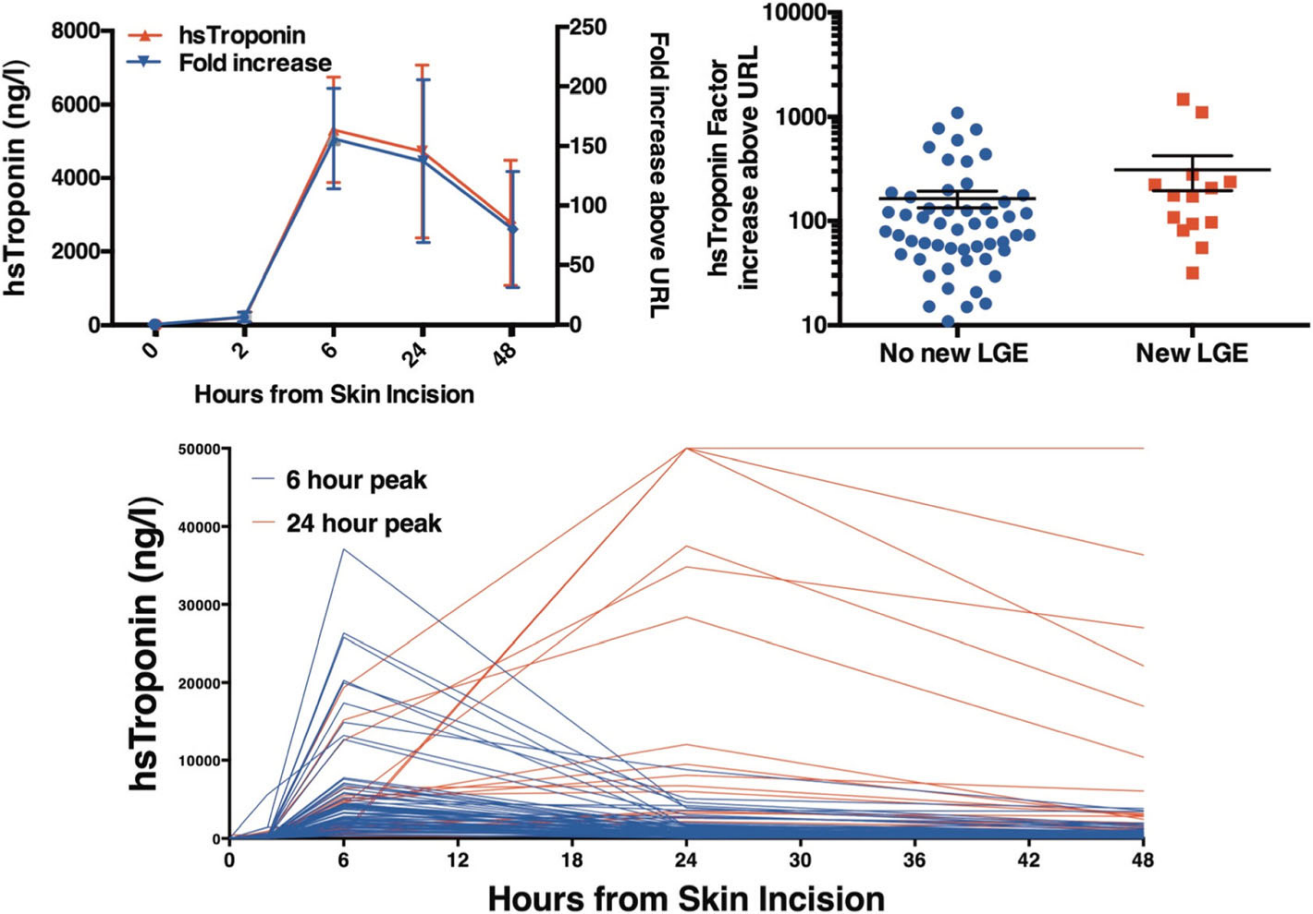
Perioperative high-sensitivity cardiac troponin. Mean ± SD and fold increase from baseline (top left). Hs-cTnI concentration fold increase above 99th centile upper reference limit (URL) according to the presence of new late-gadolinium enhancement (top right). Perioperative hs-cTnI profiles of all patients (bottom)

Complete cTnI profiles over 48 h were available in 84 patients. Plasma cTnI concentrations peaked at 6 h (median 3220 [1410 to 5610] ng/L; Fig. 4) with almost all patients demonstrating >100-fold (median 760 [151 to 1623] fold) increase from baseline to 6 h and all patients exhibiting >10-fold (median 102 [55 to 206] fold) elevation above the 99th centile URL. Patients without new LGE had lower increases (median 83 [11 to 161] fold) compared to patients with new LGE (median 174 [91 to 248] fold, *p* = 0.04; Fig. 4).

Two patterns of cTnI release were noted in the post-operative period with peak concentrations at 6 (*n* = 67) or 24 h (*n* = 18; Fig. 2). Median AUC for hs-cTnI release was 74,478 [35,100 to 164,100] ng/L/48 h. There was no correlation between cTnI AUC and cardiopulmonary bypass (*r* = 0.14, *p* = 0.11) or cross clamp time (*r* = 0.02 *p* = 0.43).

### Myocardial infarction

#### Electrocardiogram

**Fig. 5:**
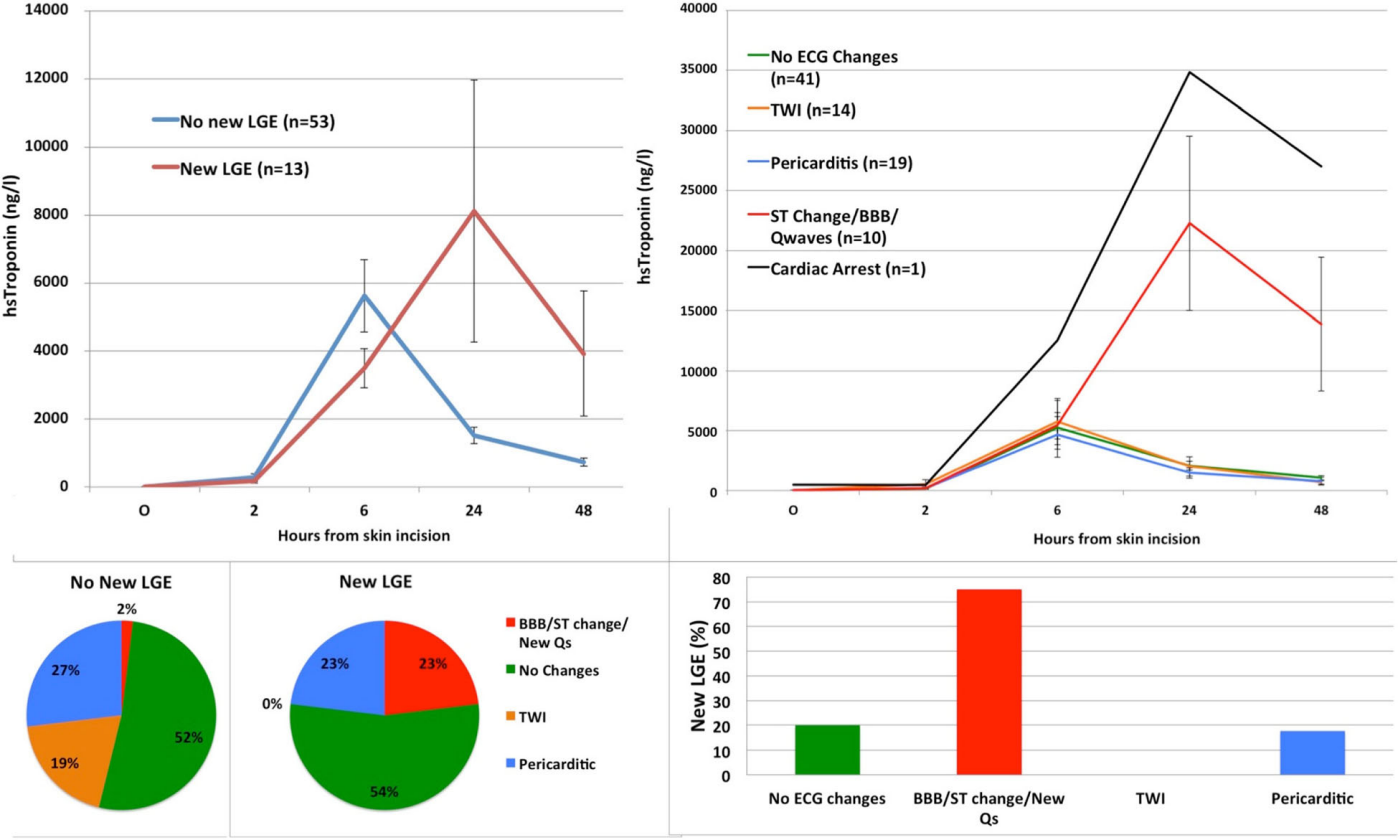
Perioperative high-sensitivity cardiac troponin concentration (mean ± SEM) according to new late gadolinium enhancement (left) and ECG changes (right). Post-operative evidence of infarction on ECG (bottom)

Eleven (13%) patients developed new ST-change/new bundle branch block/new Q-waves consistent with myocardial infarction (Fig. 5). The majority of patients had no ECG changes (*n* = 41, 48%) or non-specific pericarditic change (*n* = 19, 22%) and T wave inversion (*n* = 14, 16%). Patients with ECG evidence of infarction exhibited a cTnI peak at 24 h: 6-h mean, 6124 ng/L (95% CI 1758 to 10,490) and 24-h mean 23,410 ng/L (95% CI 8530 to 38,300). Patients without specific ECG changes of infarction (*n* = 74) had peak cTnI concentrations at 6 h: 6-h mean 5186 (95% confidence intervals 3635, 6738) ng/L and 24-h mean 1905 (95% confidence intervals 1353, 2458) ng/L.

Overall, 7/11 (64%) of patients with an ECG suggesting infarction had a rise in cTnI concentration between 6 and 24 h. In patients with no evidence of infarction on ECG, only 11/74 (15%) had a rise in cTnI concentration between 6 and 24 h. Thus ECG analysis provided a positive predictive value of a late troponin peak of 64% (95% confidence intervals 31% to 89%) and a negative predictive value of 85% (95% confidence intervals 75% to 92%). An ECG demonstrating infarction was 23% sensitive and 98% specific for the formation of LGE.

#### Cardiac magnetic resonance

Assessment of late gadolinium enhancement was available in 66 of 68 patients who underwent both pre- and post-operative CMR. Thirteen patients developed new LGE distributed by ECG category as follows; 3/4 (75%) with ST-change/new bundle branch block/new Q-waves, 7/35 (20%) with no ECG changes, 3/17 (18%) with pericarditic changes and 0/10 (0%) with T wave inversion (Fig. 5). Infarct volume by LGE correlated with AUC for cTnI (*r* = 0.33, *p* < 0.01). For left ventricular function, 8/68 (12%) of patients had at least 5% increase in ejection fraction, 20/68 (29%) had a decrease and 40/68 (59%) had no change (Additional file 2: Figure S2).

Peak plasma cTnI concentration occurred at 24 h in patients with new LGE: 6-h mean 3655 (95% confidence intervals 2228 to 5081) ng/L and 24-h mean 9073 (95% confidence intervals 5106 to 18,660) ng/L. By contrast, patients without new LGE had a peak cTnI concentration at 6 h of 5487 (95% confidence intervals 3390 to 7585) ng/L compared to a mean of 1545 (95% confidence intervals 1060 to 2031) ng/L at 24 h. Overall 6/13 (46%) of patients with new LGE had a rise in cTnI concentration between 6 and 24 h compared to 5/52 (10%) in patients without LGE (p < 0.01). A rise in troponin release after 6 h was 46% sensitive (95% CI 19 to 75) and 90% specific (95% confidence intervals 79 to 97) for detecting new LGE. The positive predictive value was 55% (95% confidence intervals 23.38 to 83.25) and the negative predictive value 87% (95% confidence intervals 75.10 to 94.63).

## Discussion

We believe this is the first in depth and detailed investigation of high-sensitivity cardiac troponin release and perioperative myocardial inflammation, injury and infarction in patients undergoing CABG surgery. This study was performed on a highly characterised and phenotyped population using the current gold standard techniques to detect myocardial inflammation, injury and infarction [11]. We have demonstrated that patients undergoing CABG surgery invariably have plasma hs-cTnI concentrations >10-fold the 99th centile that is not attributable to inflammatory or ischemic injury alone. Peri-operative type 5 myocardial infarction is often unrecognised and is associated with a delayed 24-h peak in plasma hs-cTnI concentrations.

Using the latest generation high-sensitivity assay, we have shown that cTnI release is >10-fold the upper limit of normal (99th centile) in all patients following CABG surgery. Importantly, we describe two patterns of release with an early 6-h peak and a more delayed 24-h peak. We suggest these peaks reflect perioperative myocardial injury and infarction respectively as demonstrated by late gadolinium enhancement on cardiac magnetic resonance imaging. There appears to be no single discriminatory threshold for troponin concentration that could reliably discriminate clinically overt myocardial infarction. Our data indicate that the profile, rather than peak, of troponin release may be more important when attempting to distinguish between myocardial injury and type 5 myocardial infarction.

All patients in our study exhibited at least 50-fold rise from baseline troponin and all but 4 had at least 100-fold increase. Many factors contribute to myocardial injury in the perioperative period including manipulation of the heart during on-pump surgery, cardioplegia, ischemia and patients specific factors including severity of underlying coronary artery disease and myocardial function. The average increase in cTnI concentration from baseline was nearly 2000. The third universal definition for type 5 myocardial infarction occurring during CABG surgery stipulates a cardiac marker elevation of ≥10-fold the upper limit of normal (99th centile). In our study, all patients undergoing on-pump surgery fulfil this criterion and this threshold is therefore unhelpful and has no value. This leads to the questions of whether a higher single threshold should be employed or whether alternative criteria looking at the profile of cTnI release may be more discriminatory.

Our data would not support the approach of increasing the troponin threshold to be more discriminatory. For example, increasing the threshold to >100-fold 99th centile would have excluded 64% of patients with LGE proven macro-infarction and included 43% of patients without. Our data support the findings by other groups that multiple time-point sampling to detect a late rise in troponin concentration is more discriminatory for type 5 myocardial infarction [18–20]. Selvanayagam et al. demonstrated a late peak in troponin levels in patients with new LGE compared to those without (20.3 ± 11.9 versus 11.3 ± 11.0 h) [18]. Both Pegg et al. and Lim et al. demonstrated that subjects with new LGE showed a trend for continuous increase of troponin up to 24 h whereas those without new LGE showed peaks at 6 to 12 h [19, 20]. We have here found two distinct cTnI concentration peaks at 6 and 24 h. Patients with ECG or LGE evidence of infarction were much more likely to have a peak cTnI at 24 h compared to those without. In our study, an ECG demonstrating infarction was highly specific (98%) for detecting the formation of LGE. However both ECG and cTnI profile in isolation had relatively low sensitivity for such detection. This may be because greater volume of infarction must occur in order to cause a change in the electrical or biochemical profile. We suggest that the most effective and practical method for diagnosing type 5 myocardial infarction involves cTnI testing at 6 and 24 h combined with ECG evidence of infarction. Additional use of CMR is not practical nor cost effective in the clinical setting. However, patients with a late rise in cTnI could be targeted for additional diagnostic testing such as echocardiographic imaging or angiography. Such patients may also benefit from additional anti-platelet treatment, but further studies would be required for validation.

A previous study reported new LGE in up to 78% of patients undergoing CABG surgery, but the investigators did not perform pre-operative CMR [21]. We identified LGE in 27% of pre-operative MRI scans in patients with no prior clinical history of myocardial infarction suggesting the presence of sub-clinical infarction is common even in stable patients. Consistent with our work, Pegg and colleagues also reported new LGE from preoperative scans in 8/40 (20%) of patients participating in a study of a novel hybrid method of on-pump beating heart CABG surgery [19].

Although it might have been anticipated that the magnitude of myocardial injury would correlate with CPB time, there was no association with AUC cTnI release. Furthermore there was no correlation with inflammatory cytokine release or myocardial inflammation. This suggests that injury is not mediated by inflammation alone. Other processes influence the cTnI injury such as microemboli, surgical manipulation of the heart and patient specific factors. Our data also confirm that substantial cTnI release occurs in the perioperative period without imaging evidence of infarction and new scar formation consistent with reversible myocardial injury.

We used USPIO contrast for the first time to assess in vivo myocardial inflammation following CABG surgery, similar to macrophage infiltration in patients with type 1 myocardial infarction [15]. The average pan-myocardial USPIO uptake was higher than that of healthy myocardium from controls. However it would be expected that inflammation would not be uniform throughout the heart given variation in the distribution and severity of coronary disease and surgical factors. Using the average USPIO uptake in the three segments with the highest uptake, we identified a 2-fold increase compared to the pan-myocardial values. Macrophage recruitment into the myocardium correlated weakly with hsCRP suggesting that humoral and cellular inflammation are co-ordinated. However there was no correlation with hs-cTnI or CPB time indicating that the magnitude of myocardial inflammation was not dependent on degree of myocardial injury or ischemia time. The determinants and the sequelae of higher levels of cellular inflammation post-CABG surgery require further study.

Our study has some limitations. This was a sub-analysis of the EMPIRE study [12]. In this trial, there was no demonstrable effect from the intervention (the neutrophil elastase inhibitor, elafin) on a range of clinical and surrogate outcome measures. However we cannot exclude the possibility of a weak effect on the current outcome measures. Some patients had difficulty lying flat and breath-holding in the MRI scanner for post-operative scans. This sometimes resulted in reduced scan quality. Since the USPIO-enhanced scans were carried out at later time points (5 to 14 days post-surgery), this may have hindered our ability to identify some of the correlations with our early (first 48 h) biomarker assessments.

## Conclusions

All patients undergoing CABG surgery demonstrate >10-fold elevation above the 99th centile of cardiac troponin indicating the current universal definition of type 5 myocardial infarction lacks specificity. Differing levels of myocardial inflammation post CABG surgery occurred, and did not correlate directly with the length of CPB, hs-cTnI release or circulating cytokines. A peak hs-cTnI at 6 h following CABG appears to be related to the surgical process and non-specific myocardial injury whilst a continuing increase at 24 h suggesting myocardial infarction. We would suggest cTnI sampling at 6 and 24 h post CABG surgery together with ECG assessment for the routine detection and diagnosis of type 5 myocardial infarction.

## Additional files

Additional file 1: Systemic inflammatory markers. (DOCX 387 kb)

Additional file 2: Correlations and change in ejection fraction (pre to post surgery). (DOCX 176 kb)

## Abbreviations

95% CI: 95% Confidence Interval
AUC: Area under the curve
BBB: Bundle branch block
CABG: Coronary artery bypass graft
CMR: Cardiac magnetic resonance
CPB: Cardiopulmonary bypass
CTnI: Cardiac troponin I
hsCRP: C-reactive protein by highly sensitive assay
hs-cTnI: Cardiac troponin I by highly sensitive assay
IQR: Inter-quartile range
LGE: Late gadolinium enhancement
MPO: Myeloperoxidase
SEM: Standard error of mean
TWI: T-wave Inversion
USPIO: Ultrasmall superparamagnetic particles of iron oxide
