# Supplementary figures and images for: Myocardial inflammation, injury and infarction during on-pump coronary artery bypass graft surgery
Source: J Cardiothorac Surg. 2017 Dec 16;12:115. doi: 10.1186/s13019-017-0681-6 (PMC5732376; doi:10.1186/s13019-017-0681-6)

Figure S1


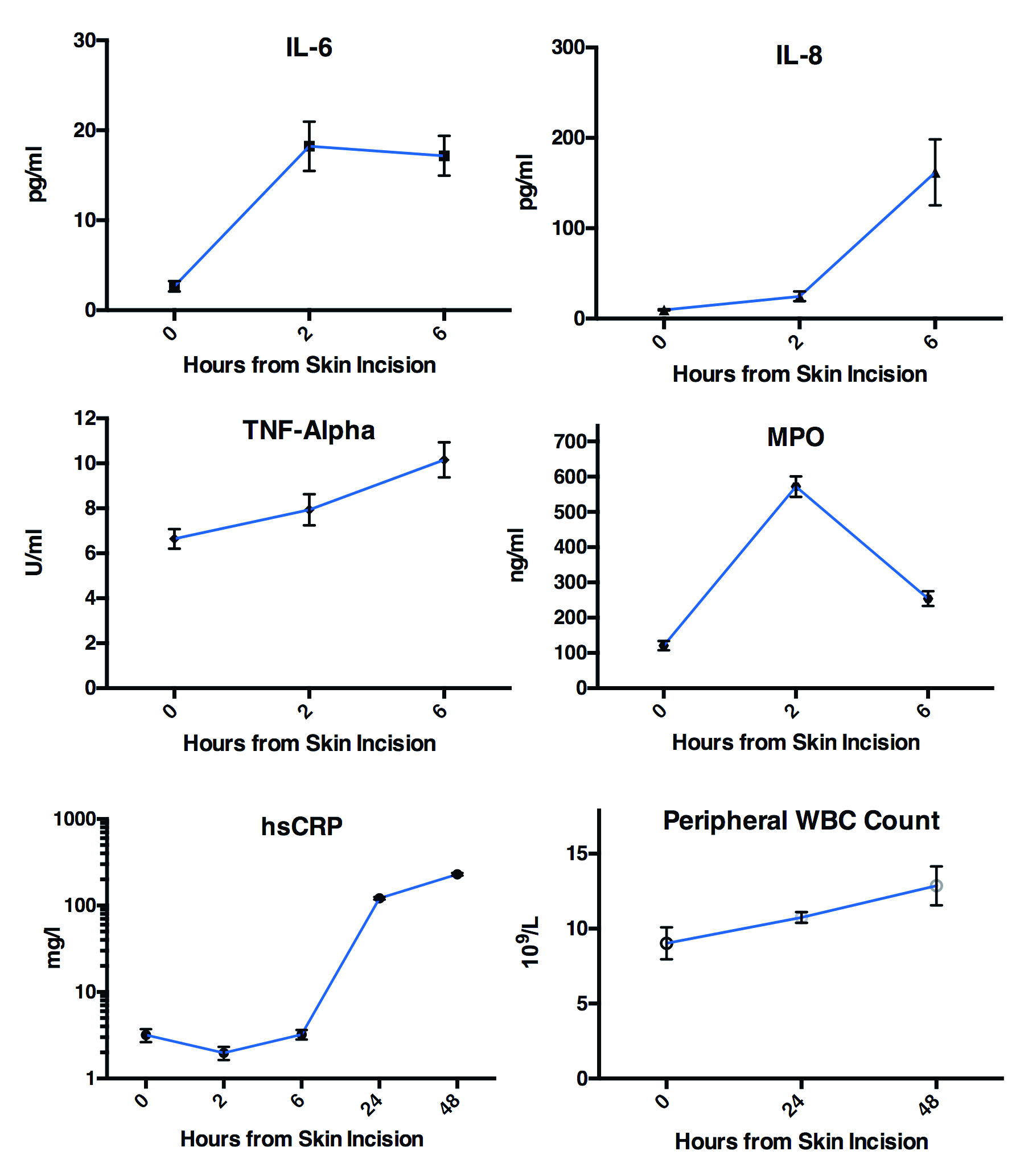

Supplement: Supplementary file 1 — Systemic inflammatory markers. (DOCX 387 kb) [file 13019_2017_681_MOESM1_ESM.docx]

Figure S2


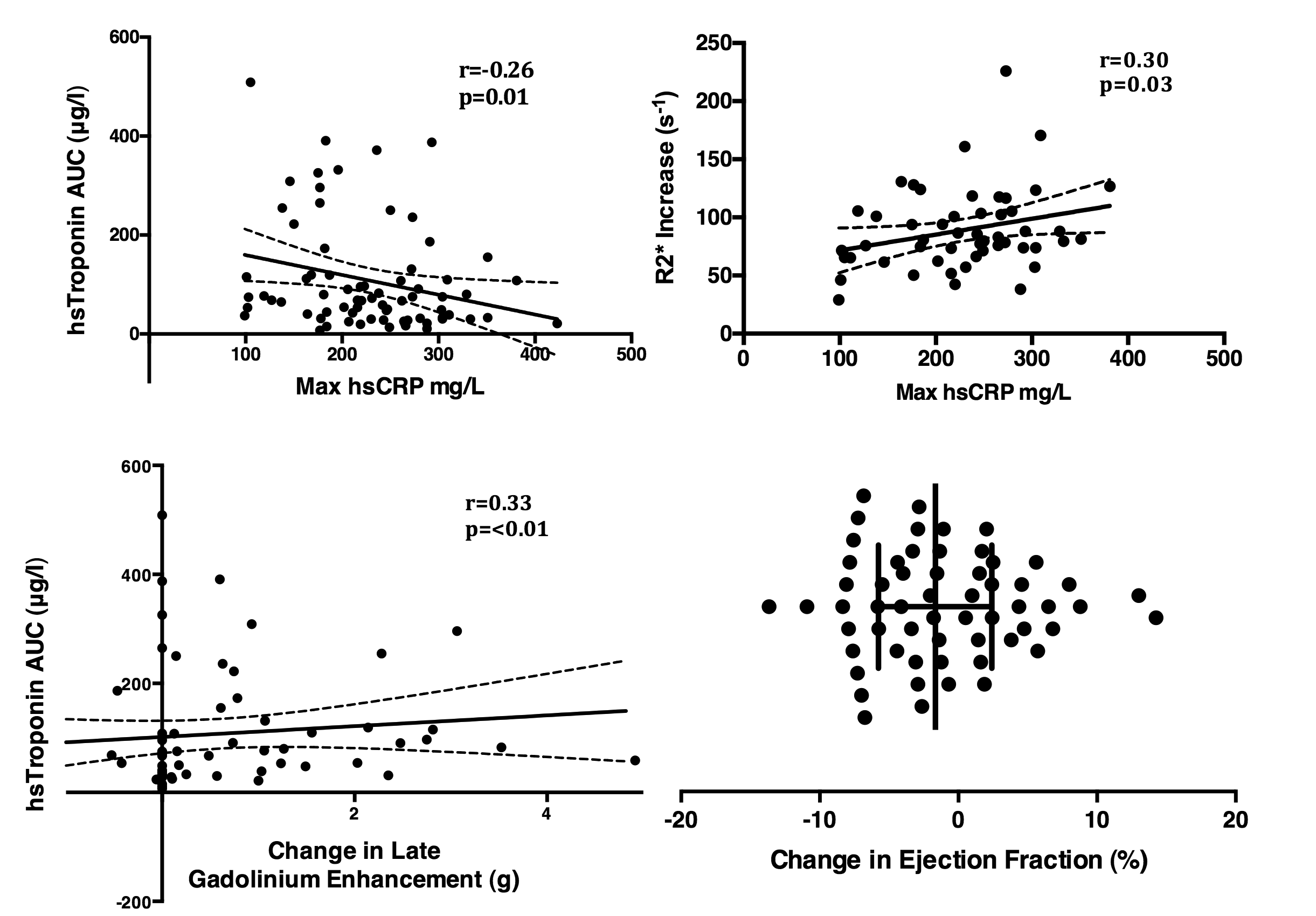

Supplement: Supplementary file 2 — Correlations and change in ejection fraction (pre to post surgery). (DOCX 176 kb) [file 13019_2017_681_MOESM2_ESM.docx]
